# Chemical disinfection as a simple and reliable method to control the amphibian chytrid fungus at breeding points of endangered amphibians

**DOI:** 10.1038/s41598-024-55946-1

**Published:** 2024-03-02

**Authors:** Barbora Thumsová, Emilio González-Miras, Ángel Rubio, Ignacio Granados, Kieran A. Bates, Jaime Bosch

**Affiliations:** 1 Asociación Herpetológica Española (AHE), Madrid, Spain; 2 Museo Nacional de Ciencias Naturales-CSIC, 28006 Madrid, Spain; 3 IMIB-Research Unit of Biodiversity (University of Oviedo, CSIC, Principality of Asturias), 33600 Mieres, Spain; 4 Agencia de Medio Ambiente y Agua de Andalucía, Consejería de Sostenibilidad, Medio Ambiente y Economía Azul, Junta de Andalucía, Seville, Spain; 5 Centro de Investigación, Seguimiento y Evaluación, Parque Nacional Sierra de Guadarrama, 28740 Rascafría, Spain; 6 Centre for Immunobiology, The Blizard Institute, Queen Mary University of London, London, E1 2AT UK

**Keywords:** Conservation biology, Herpetology, Ecology, Invasive species

## Abstract

Chytridiomycosis caused by the fungal pathogen *Batrachochytrium dendrobatidis* (*Bd*) is pushing amphibians towards extinction. Whilst mitigation methods were suggested a decade ago, we lack field trials testing their efficacy. We used the agrochemical fungicide, tebuconazole, to treat *Bd* infected breeding waterbodies of an endangered species that is highly susceptible to the fungus. Just two applications of tebuconazole led to a significant reduction in infection loads in the vast majority of sites, and at six sites the disinfection remained one/two-years post-application. Tebuconazole values drastically decreased in the waterbodies within a week after application, with no significant effects on their hydrochemical and hydrobiological characteristics. Although the use of chemicals in natural populations is undesirable, the growing existential threat to amphibians all over the world indicates that effective interventions in selected populations of endangered species are urgently needed.

## Introduction

Emerging infectious diseases are a major driver of population declines and species extinctions across the planet^1^. Disease management is increasingly necessary to reduce impacts on wild populations^2^, yet developing successful management strategies in the wild is extremely challenging and requires broad interdisciplinary collaboration among biologists, veterinarians, epidemiologists and policy makers^3^. This is especially true for parasites with a complex life cycle, free-living life stages and/or with the ability to infect multiple host-species^4^. Existing management strategies can be classified into three general groups: prevention of pathogen introduction, control of already introduced pathogens, or pathogen eradication^5^. While eradication management focuses on complete elimination of the infectious agent, control measures aim to reduce the severity of the disease impact, e.g. by minimizing transmission by reducing infection prevalence and intensity^6^, or by controlling environmental reservoirs^7^. Finally, selecting appropriate disease management in a given region and at a given time requires a thorough understanding of the cause, stage and ecology of the disease, as well as a clear identification of the risks and limitations of the method^2,5,8^.

For the past twenty years, a wealth of research has focused on developing strategies to combat the amphibian skin disease chytridiomycosis (reviewed in ref.^9^), which has been described as the worst infectious disease ever recorded among vertebrates^10^. Its causative agents, two chytrid fungi: *Batrachochytrium dendrobatidis* (*Bd*) and *Batrachochytrium salamandrivorans* (*Bsal*), have been associated with the decline and extinction of hundreds of amphibian species around the world^11^. *Bd* in particular is considered one of the most dangerous invasive species in the world and has colonized all continents where amphibians occur^12,13^. Despite the threat that *Bd* poses to native populations, the list of studies addressing ecological drivers of its emergence, or simply reporting its occurrence, strongly outweighs the limited number of disease control attempts.

Current mitigation recommendations (reviewed in refs.^14–16^) involve either environment or host manipulation, with maintenance of disease-free captive assurance colonies one of the most adopted strategies. Other promising measures include bioaugmentation with probiotics, biocontrol with aquatic predators, immunization, reduction of host abundance, manipulation of environmental temperature, increasing salinity levels or direct application of antifungals e.g. (reviewed in ref.^15^). Despite some of these methods yielding positive results under controlled laboratory conditions, attempts in the wild remain very limited.

Of the methods that have been tested in the field, in most cases results have not been satisfactory [^6^, but see ^17^]. For example, reduction of host abundance resulted in slightly lower intensity of infection only when removals were extreme^18^. Similarly, several breeding habitat interventions such as host removal, complete drying and fencing have not yielded long-term success^19^. Treatment with the antifungal drug itraconazole decreased *Bd* infection loads and increased survival of amphibians, but *Bd* load and survival of hosts returned to pre-treatment levels in less than one year. Microbiome augmentation with antifungal commensal “probiotic” bacteria has also garnered great interest as a “silver bullet” therapy, but in wild hosts, probiotic abundance declined to baseline values within one month^20^.

These unsuccessful field trials clearly suggest that without controlling the multiple pathogen reservoirs, *Bd* can never be eliminated. Indeed, tadpole removal and ex situ treatment with itraconazole followed by a combination of water draining and complete environmental chemical disinfection has been the only successful intervention^21^, with animals remaining *Bd* free and with a clear increasing trajectory in population abundance^22^. Despite this success, the environmental use of chemicals remains very controversial and total host removal is not feasible for most natural settings. The urgency of chytridiomycosis-driven declines therefore calls for rapid and less demanding interventions in time and effort that can be rolled out across different systems.

Here, we explore direct environmental application of the most widely used agrochemical fungicide, tebuconazole, as a last resort to eliminate *Bd* without previous amphibian removal. We focus on the Betic Midwife Toad (*Alytes dickhilleni*), a species endemic to the Betic mountain range of the southern Iberian Peninsula protected by national regulations (Real Decreto 139/2011) and listed as ‘endangered’ by the IUCN^23^. The species is highly susceptible to chytridiomycosis, and the rapid expansion of *Bd* throughout its entire distribution range led to subsequent mass mortality events and population extinctions^24^. Water points are scarce in the area and many have been altered for agricultural or live-stock activities, so almost 80% of the *A. dickhilleni* populations breed in small water tanks or cattle troughs (less than 100 m^3^). In 2010, 14.4% of the 111 historic populations under regular monitoring were extinct, and just 7.2% of populations held more than 1000 tadpoles^25^. Ten years later, the number of extinct populations was 34.2%, and just 1.8% of populations supported more than 1000 tadpoles, decreasing the total number of counted tadpoles from almost 36,000 to less than 14,000 (E. González-Miras, unpublished data). Here, we treated ten infected breeding waterbodies (mostly cattle troughs or small reservoirs) of *A. dickhilleni* distributed across the Andalusia region, and monitored *Bd* intensity and prevalence over time. In addition, we investigated limnological characteristics and organic matter decomposition rate in three pairs of natural ponds in central Spain, as well as characterized the skin microbiome of a subset of tadpoles from tebuconazole treated populations. We show that this mitigation approach offers a simple, cheap, and transferrable last-resort strategy that can be employed until other, more environmentally-friendly methods become available.

## Results

### Chemical disinfection of breeding waterbodies of A. dickhilleni infected with Bd

**Figure 1:**
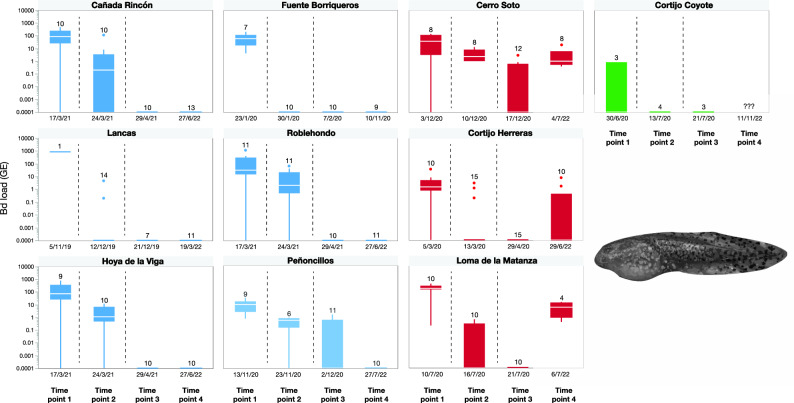
Counts of analysed *Alytes dickhilleni* individuals and *Bd* infection intensities (in genomic equivalents of zoospores and logarithmic scales) at each study site across time points (TPs): TP1, initial stage prior the chemical treatments; TP2, after the first chemical treatment; TP3, after the second chemical treatment; and TP4, one/two-year after the chemical treatments. In blue appear the sites free of *Bd* one/two-year post tebuconazole application; in red appear the sites where treatment failed, and in green appears the site where we could not corroborate successful disinfection. Dashed vertical lines indicate when treatments were implemented.

The infection load at the tebuconazole-treated sites differed significantly across time points (TPs) (F_3,354_ = 126.49, *P* < 0.001), but not across species (F_2,359_ = 1.22, *P* = 0.2967), provinces or sites (Wald tests, *P* > 0.05; Table 1). We confirmed *Bd* presence at all study sites before treatment, with an overall infection prevalence of 82% (TP1, n = 100 animals) and an average infection load for *Bd* infected individuals of 118.1 ± 199.8 GE (mean ± SD; Fig. 1). After the first treatment with tebuconazole (TP2) we detected *Bd* at eight of the ten treated sites, but the infection load significantly decreased (adjusted df = 354, t = 13.93, *P* < 0.0001). That is, 44.9% of the sampled individuals (n = 98) remained infected at TP2, with an averaged infection load of 8.5 ± 20.2 GE. After the second treatment, *Bd* was detected only at two sites with an overall prevalence of infection of 9.2% and a reduced averaged infection load with respect to TP2 of 0.9 ± 0.8 GE (t = 3.68, *P* = 0.0015). At one of these two sites, we also found *Bd* 577 days after the first tebuconazole application, and all sampled tadpoles at this site were still infected with an average infection load of 4.2 ± 6.5 GE. Moreover, we found that reinfection occurred at TP4 at another two sites where treatment was previously successful. Although the prevalence of infection at these sites was 20% and 100%, average *Bd* loads were similarly low: 4.8 ± 4.3 GE for the low-prevalence site, and 6.6 ± 6.6 GE for the high-prevalence site. We found no tadpoles at one of the tebuconazole treated sites at TP4, so we could not confirm its long-term post-treatment infection status (Fig. 1). In general, the infection load detected one/two-years later was still significantly lower than before and after the first treatment (t = 15.89, *P* < 0.0001; TP4 versus TP2, t = 2.71, *P* = 0.0351), and did not significantly differ from the values found after the second treatment (t = − 0.81, *P* = 0.8495). When considering only the three sites where the tebuconazole-treatment was unsuccessful or where the reinfection occurred, the infection load detected one/two-years post-treatment was significantly lower than the initial stage (t = 6.17, *P* < 0.0001) but only slightly higher than after the second treatment (t = -2.80, *P* = 0.0303). Finally, during the entire study period we did not detect any mass mortality outbreaks, dead individuals or any suspicious decreases in tadpole abundancies.

**Table 1:** Prevalence and apparent prevalence of infection (in parenthesis) on *Alytes dickhilleni* tadpoles at the study sites where tebuconazole was applied to reach a target concentration of 0.5 mg/L at the following time points: before mitigation (initial stage), after the first and the second treatments, and at present (1–2 years after treatments). Other amphibian species present in some sites are *Pelophylax perezi* (*Pp*) and *Salamandra salamandra* (*Ss*).

Site	Estimated water volume (m^3^)	Larvae abundance	Other species	Time point 1 (initial stage)	Time point 2 (after 1st treatment)	Time point 3 (after 2nd treatment)	Time point 4 (1–2 years after treatments)	Mitigation outcome
Cañada Rincón	675	100–200	–	90.0 (55.5–99.7)	50.0 (18.7–81.3)	0.0 (0.0–30.9)	0.0 (0.0–24.7)	Successful disinfection
Fuente Borriqueros	15	50–100	–	100.0 (59.0–100.0)	0.0 (0.0–30.9)	0.0 (0.0–30.9)	0.0 (0.0–33.6)	Successful disinfection
Lancas	25	10–50	*Ss*	100.0 (–)	14.3 (1.8–42.8)	0.0 (0.0–41.0)	0.0 (0.0–28.5)	Successful disinfection
Roblehondo	24	50–100	–	90.9 (58.7–99.8)	81.8 (48.2–97.7)	0.0 (0.0–30.9)	0.0 (0.0–28.5)	Successful disinfection
Hoya de la Viga	938	100–200	–	77.8 (40.0–97.2)	90.0 (55.5–99.8)	0.0 (0.0–30.9)	0.0 (0.0–30.9)	Successful disinfection
Peñoncillos	36	10–50	–	100.0 (66.4–100.0)	83.3 (35.9–99.6)	36.4 (10.9–69.2)	0.0 (0.0–30.9)	Successful disinfection
Cerro Soto	6	50–100	*Pp*	87.5 (47.4–99.7)	100.0 (63.1–100.0)	41.7 (15.2–72.3)	100.0 (63.1–100.0)	Unsuccessful disinfection
Cortijo Herreras	15	100–200	–	90.0 (55.5–99.8)	20.0 (4.3–48.1)	0.0 (0.0–21.8)	20.0 (2.5–55.6)	Apparent reinfection
Loma de la Matanza	80	10–50	*Pp*	100.0 (69.2–100.0)	30.0 (6.7–65.3)	0.0 (0.0–30.9)	100.0 (39.8–100.0)	Apparent reinfection
Cortijo Coyote	96	< 10	–	33.3 (0.8–90.6)	0.0 (0.0–60.2)	0.0 (0.0–70.8)	–	Possible successful disinfection

### Microbiome characteristics of threated tadpoles across populations

We found no significant differences for Shannon diversity post-tebuconazole treatment for any of the study populations (Supplementary Fig. S1). Analysis of taxa within each of the populations revealed that the majority of ASVs with mean relative abundance > 0.5% were conserved after anti-fungal treatment (Supplementary Fig. S2). Visualisation of bacterial phyla composition further showed broadly similar profiles across sampling days, however, some observable shifts in presence of key phyla were evident, suggesting that antifungal treatment may not have predictable effects on microbiome composition across populations (Supplementary Fig. S3). Given the small sample sizes for our microbiome analyses, we exercise caution in the interpretation of these results, treating them largely as qualitative with future work required.

### Changes in limnological characteristics of treated natural ponds

The tebuconazole environmental background concentration before our experiment in natural waterbodies of the Peñalara Massif Wetlands (Central Spain) was between 0.006 and 0.009 µg L^−1^, whereas after the treatments the concentrations were 120, 720 and 1260 µg L^−1^ in the treated ponds. Nevertheless, the tebuconazole concentration decreased between 99.84 and 99.99% just eight days later, reaching 0.071–0.188 µg L^−1^. Ninety-one days later, the concentration was only slightly higher in the treated ponds than in the control ponds (0.027–0.033 µg L^−1^ *versus* 0.011–0.012 µg L^−1^) and very close to the environmental background.

We found no significant differences between the treated and control ponds in terms of conductivity, dissolved oxygen, alkalinity, nitrite, nitrate, total nitrogen, total phosphorus, and chlorophyll a concentration (Supplementary Table S1). On the contrary, some parameters showed significant differences throughout the duration of the study (alkalinity, total phosphorus and chlorophyll a) but not related to the tebuconazole treatment.

Macroinvertebrate taxa richness was very similar in paired ponds, with an average of 12.0 taxa in the treated ponds compared to 11.7 in the control ponds (Supplementary Table S2). The beta diversity was very low, with a value of 0.197 (Table S3). Decomposition rates had a good fit to an exponential decay model (r^2^ = 0.51–0.94, *P* < 0.001). The slopes of the linear transformed models were significantly higher in one of the paired ponds compared to the others, however, within the same pair no differences were found between control and treated ponds (Supplementary Fig. S4).

## Discussion

Using the agrochemical fungicide tebuconazole, we successfully eliminated *Bd* at eight of the ten sites tested, and at least six remained without *Bd* one/two-years post application. Even though *Bd* was not successfully eliminated at one site, and reinfection occurred at two additional sites, our method shows promise for combatting chytridiomycosis. This approach hinges on close collaborations with environmental authorities and a thorough analysis of risks and consequences given the potential for severe off-target environmental effects^26^.

In this mitigation attempt we did not focus on managing the intensity of infection and the prevalence of infection separately. The reduction of both are important in disease management as they decrease the likelihood that a host will become infected^5^. In our system, adult specimens are usually free of *Bd* due to the high temperature and low humidity of the terrestrial environment (reviewed in ref.^9^). Conversely, *Alytes* tadpoles are usually highly infected, and the intensity of infection is strongly correlated with the prevalence of infection^24,27^. Therefore, the reduction of infection intensity of tadpoles may concurrently decrease the prevalence of infection. Indeed, after the application of tebuconazole, both measures of disease risk notably decreased or reached the zero bound, but with large confidence intervals for prevalence due to our small sample sizes (Fig. 1, Table 1). These small sample sizes do however, reflect natural population abundances, with usually no more than 50 tadpoles inhabiting small waterbodies^25^. In fact, with an overall baseline prevalence of 88% (n = 93) the desirable sample size to achieve a target population sensitivity of 99% for populations of 10 or 50–200 tadpoles would only be 5 or 6 individuals, respectively.

It is also highly possible that untreated populations close by or adult individuals of *Pelophylax perezi* inhabiting some of the treated populations could have caused the observed reinfection of Cortijo Herreras and Loma de la Matanza, or the unsuccessful disinfection of Cerro Soto. This is especially likely for Cortijo Herreras, which is very close to one untreated site that remains infected, Similarly, the other two sites where the disinfection was unsuccessful, or where reinfection occurred, support *P. perezi* populations (Table 1). The lack of total *Bd* clearance in some sites warrants further consideration and concern, with a prior study demonstrating poorer host health outcomes with itraconazole treatment (another triazole fungicide) when *Bd* was not completely eliminated or if reinfection occurred^28^. In any case, our results indicate that direct tebuconazole application without removing tadpoles appears to be more effective than the approach used by Bosch et al.^21^ and, undoubtedly, significantly less demanding and much cheaper.

Although some studies have shown tebuconazole to be highly persistent in the environment^29^, our experiment performed in the Peñalara Massif Wetlands found the concentration of tebuconazole fell to close to background concentrations only a week after application. The expected initial tebuconazole concentration in treated ponds was lower in one case (25%), but clearly higher in two of them (14–250%), due to the difficulty of accurately measuring the volume of water in natural ponds with irregular contours and depths. This suggests that even slightly higher values than those established are suitable for amphibian treatment and have little obvious environmental effects in the short to medium term. Moreover, our experiment indicates the potential suitability of the method in more natural waterbodies under exceptional circumstances. No hydrochemical differences were found between treated and control ponds, and the hydrochemical characteristics were within the typical values found in the Peñalara Massif Wetlands. Significant changes in alkalinity, total phosphorous and chlorophyll with time could be explained as a consequence of phytoplankton growth and water volume reduction throughout summer in all ponds^30^, suggesting a lack of effect of the fungicide on phytoplankton. The invertebrate and particularly macroinvertebrate communities did not show any differences between treated and control ponds and also showed similar species richness and taxa compositions as others found in these wetlands^30^. We found no effect on zooplankton (Table S4), although only one of the paired ponds was studied, limiting our ability to draw conclusions.

A key consideration was whether the addition of fungicide could have a discernible effect on organic matter decomposition rate, since it is well known that this ecological process is largely dependent on fungal taxa in aquatic ecosystems^31^. Our results show that decomposition rate is not affected by tebuconazle exposure, as the organic matter degradation is similar between control and treated ponds in all groups, regardless of differences between groups of ponds attributable to environmental conditions such as hydroperiod, turnover rate, organic matter content, etc. Furthermore, our preliminary microbiome analysis indicated no significant changes in skin bacterial diversity after tebuconazole treatment, although compositional differences were qualitatively observed. Additional investigations of the effects of tebuconazole treatment on aquatic bacterial and fungal communities are therefore of paramount importance to properly ascertain the environmental risks of this mitigation strategy.

Aquatic ecosystems are known to be threatened by pesticides and fertilizers worldwide^32^. Tebuconazole, is the most widely used product, and is commonly detected in waterbodies within agricultural landscapes^33^. Like other azoles, tebuconazole can indirectly impair amphibian survival, development and metamorphic traits, or produce severe irreversible injury to multiple organs, as well as promote resistance or tolerance in opportunistic fungi^34–36^. Despite these concerns, waterbodies in agricultural catchments are exposed to tebuconazole repeatedly^37^, and all available research on biological responses refer to chronic laboratory trials. Our aim is not to endorse the widespread application or repeated exposure of tebuconazole, but to determine a minimal ecologically invasive combination of applications and dosages in a very limited number of sites with endangered species, to promote effective disease control. In any case, candidate sites to be treated should be artificial sites, even created intentionally for the reproduction of the target species and, always, isolated places inaccessible to humans and livestock.

Whether amphibians can survive the ongoing global-scale decline will depend on the development of an effective strategy to prevent their disappearance locally and globally. When dealing with the last remaining populations of a threatened species, it is vital to consider how closely sub-optimal strategies align with the best-practice guidelines to promote positive ethical, evolutionary, epidemiological and environmental outcomes^2^. Despite our promising results, we insist that each chemical intervention should be implemented with caution, well timed and rigorously monitored for adverse events across short and long temporal scales. The urgency of the global demise of amphibians compels us to intervene now, because extinction is forever.

## Methods

All applicable institutional and/or national guidelines for the care and use of animals were followed. The animal research complied with the ARRIVE guidelines and was approved by the animal ethics committee of CSIC (reference number 666/2018).

### Chemical disinfection of sites with *Bd*

**Figure 2:**
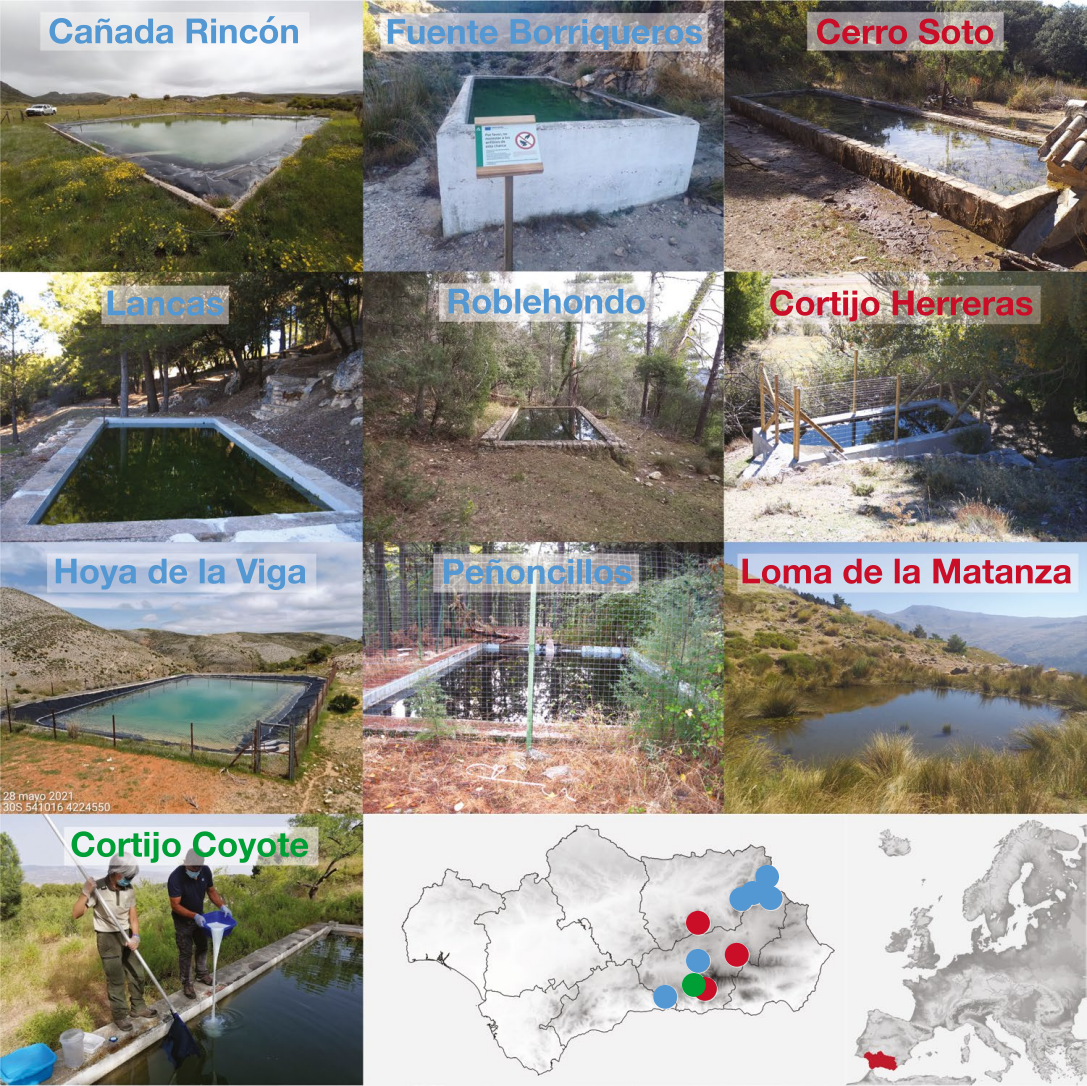
*Alytes dickhilleni* populations treated with tebuconazole into the Andalusia region. In blue are sites free of *Bd* one/two-year post-application, in red sites where treatment failed or re-infection occurred, and in green appears the site where we could not corroborate successful disinfection two years later. Map generated using QGIS 3.16 (https://qgis.org/).

We tested whether tebuconazole could be used to combat *Bd* in natural populations of highly susceptible *A. dickhilleni*^24^. We focused on larval stages since the *Alytes* genus is highly terrestrial and in dry temperate zones the adults are mostly free of *Bd*^9^. We selected a total of ten waterbodies (mostly cattle troughs or small reservoirs) distributed across the Andalusia region (Fig. 2).

We applied the tebuconazole-based fungicide DARCOS® (tebuconazole, 250 g. L^−1^) twice, without previous tadpole removal to reach a final concentration of 500 µg L^−1^. We chose this concentration based on being the highest that gave 100% host survival in a pilot experiment with tenfold serial dilutions with pond water from 500,000 to 5 µg L^−1^ in six 250 mL tanks each containing five tadpoles in early development stages. We thoroughly mixed the tebuconazole to assure that the chemical was evenly dissolved, and checked all treated sites during the study period on a monthly basis.

Depending on tadpole abundance, we collected up to 15 samples per site by swabbing tadpole’s mouthparts. Sampling was conducted at four different time points (TPs): (1) immediately before the first tebuconazole application, (2) around 1–5 weeks after the first application and immediately before the second application, (3) around 2–8 weeks after the second application, and (4) around one/two-years after applications. When *A. dickhilleni* was scarce, we additionally sampled tadpoles of other species (n = 9). At each site, we further skin-swabbed two *A. dickhilleni* tadpoles to characterize their microbial diversity across treatment stages.

We did not include tebuconazole unexposed control sites because even in low elevation areas the prevalence of infection of *Alytes* tadpoles remains close to 100% for most of the year^27^. Spontaneous *Bd* clearance has also never been observed in any of the 15 study over the last 15 years (Supplementary Fig. S5).

We processed *Bd* swabs according to Boyle et al.^38^ with negative and positive controls with known concentrations of zoospore genomic equivalents (GE) (from 0.1 to 10,000 GE).

### Effect of tebuconazole on the bacterial microbiome of tadpoles

We extracted microbial genomic DNA from skin swabs using the DNeasy Blood and Tissue Kit (Qiagen) and submitted the microbial genomic DNA to Dalhousie University’s Integrated Microbiome Resource (imr.bio) for the amplification of the V3-V4 region of the 16S rRNA gene. We performed microbiome sequence processing using DADA2 v1.25.0^39^ to infer amplicon sequence variants (ASVs). Reads were filtered at the appearance of a quality score of two or lower and excluded forward and reverse reads of 280 and 200 bases or less respectively. Reads containing any non-assigned bases, or an expected error rate above two were also excluded. ASVs were computed and paired reads were merged into single consensus reads. Chimeric sequences were removed from the dataset and ASVs were then assigned taxonomy using the naïve Bayesian classifier algorithm, with the silva database v138.1^40^. Reads assigned as chloroplast and unclassified Phyla were removed prior to downstream processing.

Since our study design did not include a tebuconazole unexposed control, we were unable to disentangle the effects of antifungal treatment from temporal changes in the bacterial microbiome. We therefore consider our bacterial microbiome analysis as exploratory. We focused our analyses on populations with successfully sequenced bacterial microbiome samples across at least two time points and with a minimum of two samples per time point, which yielded a total of six populations (26 samples). For alpha diversity analyses, we analysed each population separately. To mitigate the effects of uneven read depths across samples, we rarefied our dataset to the lowest read depth sample for each population. We calculated Shannon diversity using Phyloseq^41^ and used a t-test or ANOVA to examine whether alpha diversity differed significantly between visits. To examine changes in presence/absence of taxa over time for each population, we filtered each population to include ASVs with a mean relative abundance > 0.5% and identified common taxa across the sampling periods using UpSetR^42^. We further visualized microbiome compositional changes over time for Phyla (mean taxa relative abundance > 0.5%) for each population using stacked bar plots generated using the Microbiome package in R^43^.

### Effect of tebuconazole on natural waterbodies

We tested the effect of tebuconazole application on several limnological characteristics in three pairs of temporary ponds free of amphibians in the Peñalara Massif Wetlands, the index locality of *Bd* in Europe (Sierra de Guadarrama National Park, Central Spain), ranging from 17 to 70 m^2^ in area and 0.25 to 0.55 m in depth. The ponds in each pair were between 5 and 20 m apart, while the pairs of ponds were between 110 and 410 m apart. On June 21, 2021 we applied the fungicide to all experimental ponds (randomly assigned) to reach a concentration of 500 µg L^−1^. We collected water samples immediately after the treatment and repeated 8 and 22 days later, as well as 91 days later following the filling of the ponds after the dry period. Then we measured dissolved oxygen and conductivity in situ with a multiprobe sonde.

Macroinvertebrates were sampled 23 days after tebuconazole addition using a 250 µm sampling net (across 3 pond pairs), and zooplankton were collected with a 100 µm net (for 1 pond pair). We used the Decomposition and Consumption Tablets, DECOTABs^44^, inside a 400 µm mesh bag to avoid macroinvertebrate consumption, to measure the possible effect of tebuconazole on the natural decomposition rate. We removed five DECOTABS from each pond during following visits up to 31–62 days later, depending on the pond hydroperiod, and weighed them to obtain the remaining mass percentage. We performed hydrochemical analyses following standard methods^45^.

To look for significant differences in the limnological characteristics of natural waterbodies between the sampling dates (time), between the application of tebuconazole (treatment) or the interaction of these two factors we used a repeated measures analysis of variance (RM-ANOVA). To determine the possible effect of tebuconazole on the natural decomposition rate we transformed the exponential decay model of weight with respect to time to a linear relationship and tested the slopes differences with EMMEANS package.

### Data analyses

We calculated prevalence as the proportion of *Bd* positive individuals over the total sampled for each site at a particular time point. Using Epitools^46^ we calculated apparent prevalence of infection, and the required sample size at each site to achieve a target population sensitivity of 99% (test sensitivity: 0.9; test specificity: 0.99; confidence levels: 0.95; Clopper-Pearson exact method). We analyzed differences in *Bd* load across TPs using a general linear mixed model and Tukey’ tests on X + 1 log10-transformed infection loads. Site was nested within province as a random effect and species identity was a fixed effect.

### Supplementary Information

Supplementary Information.

## Data Availability

Raw data is provided at the Supplementary information.
