# Author Correction: Chemical disinfection as a simple and reliable method to control the amphibian chytrid fungus at breeding points of endangered amphibians

**DOI:** 10.1038/s41598-024-57246-0

**Published:** 2024-03-20

**Authors:** Barbora Thumsová, Emilio González-Miras, Ángel Rubio, Ignacio Granados, Kieran A. Bates, Jaime Bosch

**Affiliations:** 1 Asociación Herpetológica Española (AHE), Madrid, Spain; 2 Museo Nacional de Ciencias Naturales-CSIC, 28006 Madrid, Spain; 3 IMIB-Research Unit of Biodiversity (University of Oviedo, CSIC, Principality of Asturias), 33600 Mieres, Spain; 4 Agencia de Medio Ambiente y Agua de Andalucía, Consejería de Sostenibilidad, Medio Ambiente y Economía Azul, Junta de Andalucía, Seville, Spain; 5 Centro de Investigación, Seguimiento y Evaluación, Parque Nacional Sierra de Guadarrama, 28740 Rascafría, Spain; 6 Centre for Immunobiology, The Blizard Institute, Queen Mary University of London, London, E1 2AT UK

Correction to: *Scientific Reports* 10.1038/s41598-024-55946-1, published online 02 March 2024

The Acknowledgments section in the original version of this Article was omitted. The Acknowledgments section now reads:

“Special thanks to Jesús del Río, coordinator of the conservation program of endangered amphibians of Andalusia, and to Juan A. Vielva, director of CISE-Sierra de Guadarrama NP. BT was supported by ‘Doctorados Industriales de la Comunidad de Madrid’ (Ref. IND2020/AMB-17438). This study was funded by Organismo Autónomo Parques Nacionales of Spain (Project Ref. 2399/2017, PI: JB). KB was funded by an E.P. Abraham Junior Research Fellowship from St Hilda’s College Oxford.”

The original Article has been corrected.

