# Supplementary material for: Chemical disinfection as a simple and reliable method to control the amphibian chytrid fungus at breeding points of endangered amphibians
Source: Sci Rep. 2024 Mar 2;14:5151. doi: 10.1038/s41598-024-55946-1 (PMC10908824; doi:10.1038/s41598-024-55946-1)
Supplement: Supplementary file 1 — Supplementary Information. [file 41598_2024_55946_MOESM1_ESM.docx]

**Supplementary information**

**Chemical disinfection as a simple and reliable method to control the amphibian chytrid fungus on breeding points of endangered amphibians**

Table S1. Hydrochemistry of control and treated ponds.

**Temperature Conductivity Oxigen Alcalinity Nitrate Nitrite Total Total Chlorophyll a**

**Date Pond Treatment (°C) (µS/cm) saturation (%) (µeq/l) (µg/l) Nitrate (µg/l) Nitrogen (µg/l) Phosphorus (µg/l) (µg/l)**

21/6/21 XIX-07 Control 8.5 4 107.5 16 2 3 186 0 1.75

21/6/21 XIX-12 Treated 8.9 4 110 24 28 1 99 1 1.31

21/6/21 XVI-01 Control 11.2 9 111 20 0 7 181 0 1.9

21/6/21 XVI-10 Treated 11.9 8 110.8 36 5 1 466 1 0.48

21/6/21 XXIV-05 Control 7.5 19 106.5 24 6 3 170 0 0.54

21/6/21 XXIV-03 Treated 7.4 12 108.5 16 6 1 225 2 0.51

29/6/21 XIX-07 Control 11 4 107.5 40 4 1 142 2 0.85

29/6/21 XIX-12 Treated 12.8 3 111.2 52 6 1 66 2 0.6

29/6/21 XVI-01 Control 15.7 14 106.2 48 3 1 235 4 1.75

29/6/21 XVI-10 Treated 17.1 8 110.8 48 4 1 657 12 0.56

29/6/21 XXIV-05 Control 11.8 9 101.8 36 5 3 263 10 0.79

29/6/21 XXIV-03 Treated 11.9 6 106 36 9 2 449 7 1.13

13/7/21 XIX-07 Control 12.7 3 105.2 44 9 3 203 25 1.93

13/7/21 XIX-12 Treated 12.8 5 103.9 32 10 4 290 35 3.34

13/7/21 XVI-01 Control 15.3 11 115.2 56 5 2 301 11 1.92

13/7/21 XVI-10 Treated 15.8 12 118.1 36 6 1 816 19 2.13

13/7/21 XXIV-05 Control 11.2 19 107.8 68 6 1 570 24 1.87

13/7/21 XXIV-03 Treated 11.2 10 109.4 48 7 1 1090 42 3.92

Table S2. Macroinvertebrate individuals per unit effort in control (C) and treated (T) ponds on 13 July 2021.

|  | Order | Family | Species | XIX-07 (C) | XIX-12 (T) | XVI-01 (C) | XVI-10 (T) | XXIV-05 (C) | XXIV-03 (T) |
| --- | --- | --- | --- | --- | --- | --- | --- | --- | --- |
| **Bivalvia** | | | | | | | | | |
|  | Veneroida | | | | | | | | |
|  |  | Sphaeriidae | | | | | | | |
|  |  |  | *Pisidium casertanum^1^* | 46 | 38 | 1 |  | 44 |  |
| **Oligochaeta** | | | | | | | | | |
|  |  |  | Oligochaeta indet*^1^* |  | 16 | 1 |  | 16 | 2 |
| **Insecta** | | | | | | | | | |
|  | Coleoptera | | | | | | | | |
|  |  | Dytiscidae | | | | | | | |
|  |  |  | *Agabus nebulosus^2^* | 2 | 1 | 4 | 1 |  | 1 |
|  |  |  | *Agabus bipustulatus^2^* | 5 |  |  |  |  | 9 |
|  |  |  | *Agabus* sp.^1^ |  |  |  |  |  | 1 |
|  |  |  | *Dytiscus marginalis^1,2^* |  |  |  | 2 |  | 2 |
|  |  |  | *Dytiscus* sp. *^1^* |  | 6 | 1 |  | 1 |  |
|  |  |  | *Hydroporus* sp. *^1,2^* |  | 2 | 3 |  |  | 1 |
|  |  | Elmidae | | | | | | | |
|  |  |  | *Oulimnius* sp.*^1^* |  | 3 |  |  |  |  |
|  |  | Helophoridae | | | | | | | |
|  |  |  | *Helophorus* sp. *^2^* | 5 | 14 | 9 | 16 | 4 | 16 |
|  |  | Hydrophilidae | | | | | | | |
|  |  |  | *Helochares* sp. *^2^* |  |  |  | 1 | 1 |  |
|  |  |  | Hydrophilidae indet. *^1^* |  | *^2^* |  | 2 |  |  |
|  | Diptera | | | | | | | | |
|  |  | Chironomidae | | | | | | | |
|  |  |  | Chironomidae indet. *^1^* | 208 | 264 | 264 | 96 | 210 | 224 |
|  |  | Culicidae | | | | | | | |
|  |  |  | *Culex* sp. *^1^* |  |  |  |  |  | 16 |
|  | Ephemeroptera | | | | | | | | |
|  |  | Baetidae | | | | | | | |
|  |  |  | *Cloeon dipterum^1^* |  | 1 |  |  | 1 |  |
|  | Heteroptera | | | | | | | | |
|  |  | Corixidae | | | | | | | |
|  |  |  | *Micronecta* sp. *^1^* | 1 | 2 |  |  |  |  |
|  |  |  | *Sigara nigrolineata^2^* |  |  | 1 |  | 1 |  |
|  |  |  | *Sigara* sp. *^1,2^* | 2 | 1 | 24 |  |  |  |
|  |  |  | Corixidae indet. *^1^* |  |  | 28 | 48 | 44 | 28 |
|  |  | Gerridae | | | | | | | |
|  |  |  | *Gerris thoracicus^2^* |  |  |  | 2 | 3 |  |
|  |  |  | *Gerris* sp. *^1^* | 1 |  |  |  |  |  |
|  |  | Notonectidae | | | | | | | |
|  |  |  | *Notonecta glauca^2^* | 1 |  | 1 |  |  |  |
|  |  |  | *Notonecta maculata^2^* |  | 1 | 1 |  |  | 2 |
|  |  |  | *Notonecta* sp. *^1^* | 1 | 2 | 18 | 2 |  | 2 |
|  | Odonata | | | | | | | | |
|  |  | Libellulidae | | | | | | | |
|  |  |  | *Sympetrum* sp. *^1^* |  |  | 3 | 10 | 1 | 2 |

Table S3. Macroinvertebrate alpha and beta diversity in control (C) and treated (T) ponds.

**Pond Shannon alpha diversity index**

XIX-07 (C) 0.807

XIX-12 (T) 0.843

XVI-01 (C) 1.049

XVI-10 (T) 1.311

XXIV-05 (C) 1.012

XXIV-03 (T) 1.043

**Paired ponds beta diversity**

XIX-07 (C) XXIV-05 (C) 0.070

XIX-07 (C) XXIV-03 (T) 0.128

XIX-07 (C) XVI-10 (T) 0.216

XIX-07 (C) XVI-01 (C) 0.123

XIX-07 (C) XIX-12 (T) 0.029

XIX-12 (T) XXIV-05 (C) 0.076

XIX-12 (T) XXIV-03 (T) 0.112

XIX-12 (T) XVI-10 (T) 0.189

XIX-12 (T) XVI-01 (C) 0.106

XVI-01 (C) XXIV-05 (C) 0.101

XVI-01 (C) XXIV-03 (T) 0.069

XVI-01 (C) XVI-10 (T) 0.103

XVI-10 (T) XXIV-05 (C) 0.105

XVI-10 (T) XXIV-03 (T) 0.086

XXIV-03 (T) XXIV-05 (C) 0.103

Table S1. Zooplankton individuals per unit effort in in control (C) and treated (T) ponds on 13 July 2021.

**Species XVI-01 (C) XVI-10 (T)**

*Ceriodapnia reticulata* 640 2925

*Chidorus sphaericus* 24 -

*Tropocyclops prasinus* 8 194

*Chirocephalus diaphanus* - 6

Figure S1. Shannon diversity of bacterial taxa over time for selected study populations. Visit 1 corresponds to the initial stage prior the tebuconazole treatments; Visit 2, after the first tebuconazole treatment; and Visit 3 after the second tebuconazole treatment. In blue are labeled sites free of *Bd* one/two-year post-application, and in red sites where treatment failed or where re-infection occurred.

Figure S2. Changes in presence/absence of microbiome taxa over time for selected study populations. Bacterial taxa with a mean relative abundance > 0.5% were considered. Visit 1 corresponds to the initial stage prior the tebuconazole treatments; Visit 2, after the first tebuconazole treatment; and Visit 3 after the second tebuconazole treatment. In blue are labeled sites free of *Bd* one/two-year post-application, and in red sites where treatment failed or where re-infection occurred.


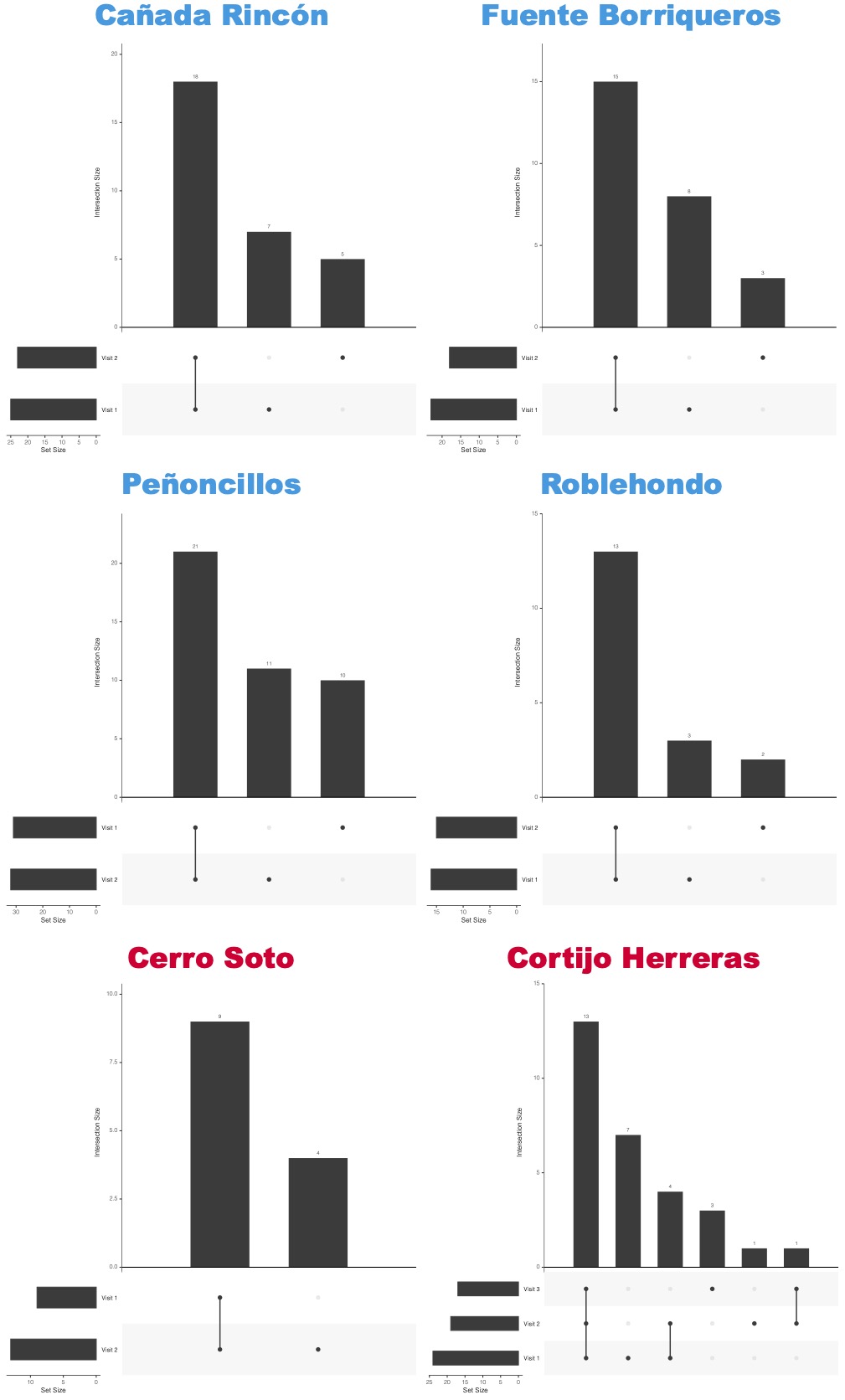


Figure S3. Phyla composition of bacterial taxa. Visit 1 corresponds to the initial stage prior the tebuconazole treatments; Visit 2, after the first tebuconazole treatment; and Visit 3 after the second tebuconazole treatment. In blue are labeled sites free of *Bd* one/two-year post-application, and in red sites where treatment failed or where re-infection occurred.

Figure S4. Linear regression of the percentage of remaining DECOTABs against time in the different ponds.


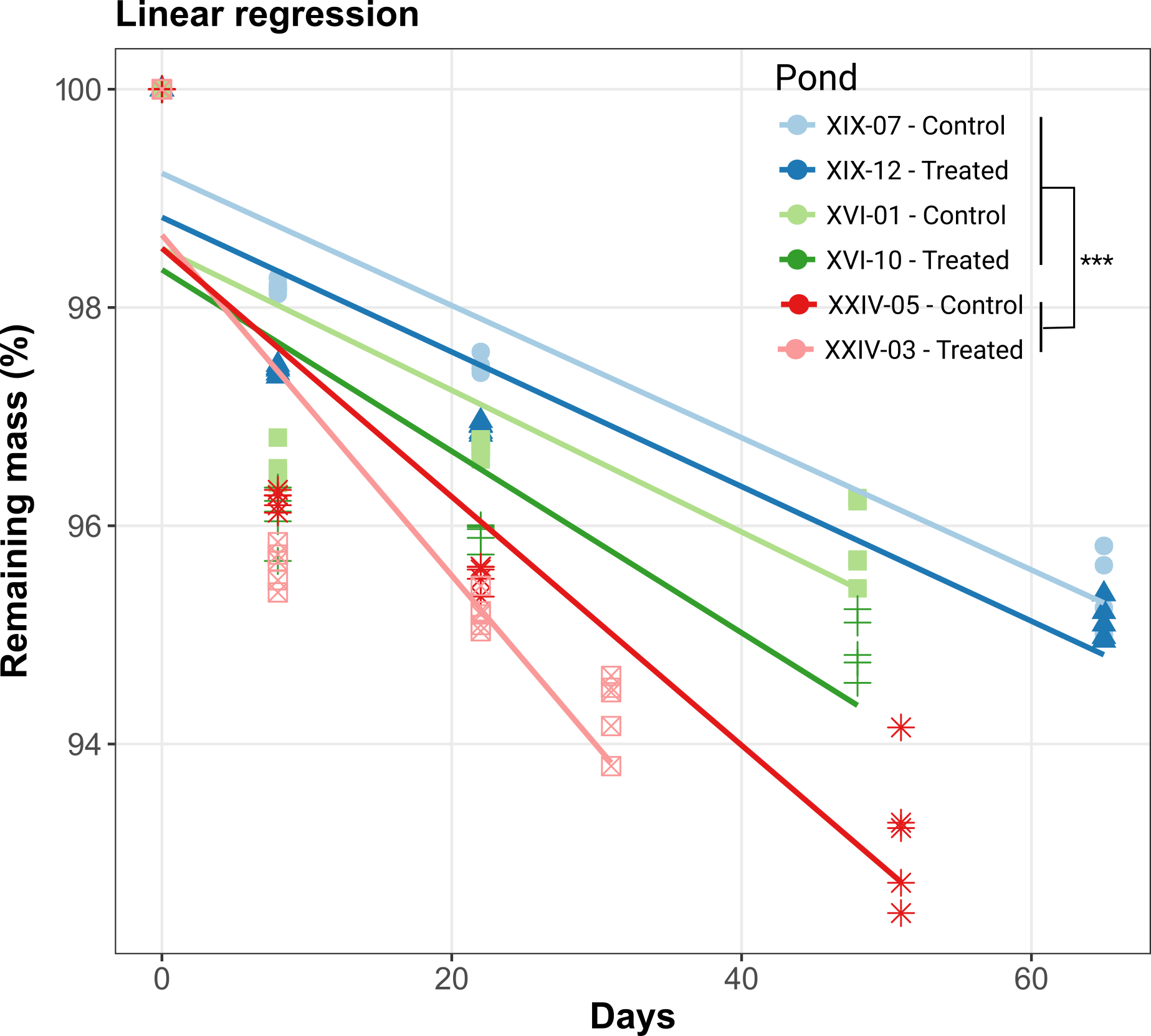


Figure S5. Prevalence of infection of *Alytes* *dickhilleni* in the 15 populations subjected to *Bd* monitoring over the last 15 years when sampling events were grouped into three periods of time (initial, medium, present) after the first *Bd* positive record was recorded at each population.

**Raw data**

| **Time point** | **Date** | **Site** | **Province** | **Specie** | **Bd load** |
| --- | --- | --- | --- | --- | --- |
| TP1 | 17/03/2021 | Cañada Rincón | Jaén | Alytes dickhilleni | 81.6 |
| TP1 | 17/03/2021 | Cañada Rincón | Jaén | Alytes dickhilleni | 181.6 |
| TP1 | 17/03/2021 | Cañada Rincón | Jaén | Alytes dickhilleni | 67.9 |
| TP1 | 17/03/2021 | Cañada Rincón | Jaén | Alytes dickhilleni | 85 |
| TP1 | 17/03/2021 | Cañada Rincón | Jaén | Alytes dickhilleni | 14.3 |
| TP1 | 17/03/2021 | Cañada Rincón | Jaén | Alytes dickhilleni | 405.4 |
| TP1 | 17/03/2021 | Cañada Rincón | Jaén | Alytes dickhilleni | 30.2 |
| TP1 | 17/03/2021 | Cañada Rincón | Jaén | Alytes dickhilleni | 470.2 |
| TP1 | 17/03/2021 | Cañada Rincón | Jaén | Alytes dickhilleni | 114.3 |
| TP1 | 17/03/2021 | Cañada Rincón | Jaén | Alytes dickhilleni | 0 |
| TP2 | 24/03/2021 | Cañada Rincón | Jaén | Alytes dickhilleni | 2 |
| TP2 | 24/03/2021 | Cañada Rincón | Jaén | Alytes dickhilleni | 0.5 |
| TP2 | 24/03/2021 | Cañada Rincón | Jaén | Alytes dickhilleni | 0 |
| TP2 | 24/03/2021 | Cañada Rincón | Jaén | Alytes dickhilleni | 113.1 |
| TP2 | 24/03/2021 | Cañada Rincón | Jaén | Alytes dickhilleni | 0 |
| TP2 | 24/03/2021 | Cañada Rincón | Jaén | Alytes dickhilleni | 0 |
| TP2 | 24/03/2021 | Cañada Rincón | Jaén | Alytes dickhilleni | 0 |
| TP2 | 24/03/2021 | Cañada Rincón | Jaén | Alytes dickhilleni | 0 |
| TP2 | 24/03/2021 | Cañada Rincón | Jaén | Alytes dickhilleni | 8.4 |
| TP2 | 24/03/2021 | Cañada Rincón | Jaén | Alytes dickhilleni | 0.4 |
| TP3 | 29/04/2021 | Cañada Rincón | Jaén | Alytes dickhilleni | 0 |
| TP3 | 29/04/2021 | Cañada Rincón | Jaén | Alytes dickhilleni | 0 |
| TP3 | 29/04/2021 | Cañada Rincón | Jaén | Alytes dickhilleni | 0 |
| TP3 | 29/04/2021 | Cañada Rincón | Jaén | Alytes dickhilleni | 0 |
| TP3 | 29/04/2021 | Cañada Rincón | Jaén | Alytes dickhilleni | 0 |
| TP3 | 29/04/2021 | Cañada Rincón | Jaén | Alytes dickhilleni | 0 |
| TP3 | 29/04/2021 | Cañada Rincón | Jaén | Alytes dickhilleni | 0 |
| TP3 | 29/04/2021 | Cañada Rincón | Jaén | Alytes dickhilleni | 0 |
| TP3 | 29/04/2021 | Cañada Rincón | Jaén | Alytes dickhilleni | 0 |
| TP3 | 29/04/2021 | Cañada Rincón | Jaén | Alytes dickhilleni | 0 |
| TP4 | 27/06/2022 | Cañada Rincón | Jaén | Alytes dickhilleni | 0 |
| TP4 | 27/06/2022 | Cañada Rincón | Jaén | Alytes dickhilleni | 0 |
| TP4 | 27/06/2022 | Cañada Rincón | Jaén | Alytes dickhilleni | 0 |
| TP4 | 27/06/2022 | Cañada Rincón | Jaén | Alytes dickhilleni | 0 |
| TP4 | 27/06/2022 | Cañada Rincón | Jaén | Alytes dickhilleni | 0 |
| TP4 | 27/06/2022 | Cañada Rincón | Jaén | Alytes dickhilleni | 0 |
| TP4 | 27/06/2022 | Cañada Rincón | Jaén | Alytes dickhilleni | 0 |
| TP4 | 27/06/2022 | Cañada Rincón | Jaén | Alytes dickhilleni | 0 |
| TP4 | 27/06/2022 | Cañada Rincón | Jaén | Alytes dickhilleni | 0 |
| TP4 | 27/06/2022 | Cañada Rincón | Jaén | Alytes dickhilleni | 0 |
| TP4 | 27/06/2022 | Cañada Rincón | Jaén | Alytes dickhilleni | 0 |
| TP4 | 27/06/2022 | Cañada Rincón | Jaén | Alytes dickhilleni | 0 |
| TP4 | 27/06/2022 | Cañada Rincón | Jaén | Alytes dickhilleni | 0 |
| TP1 | 03/12/2020 | Cerro Soto | Jaén | Alytes dickhilleni | 10.4 |
| TP1 | 03/12/2020 | Cerro Soto | Jaén | Alytes dickhilleni | 0 |
| TP1 | 03/12/2020 | Cerro Soto | Jaén | Alytes dickhilleni | 21.8 |
| TP1 | 03/12/2020 | Cerro Soto | Jaén | Alytes dickhilleni | 143.6 |
| TP1 | 03/12/2020 | Cerro Soto | Jaén | Alytes dickhilleni | 86.5 |
| TP1 | 03/12/2020 | Cerro Soto | Jaén | Alytes dickhilleni | 53 |
| TP1 | 03/12/2020 | Cerro Soto | Jaén | Alytes dickhilleni | 119.2 |
| TP1 | 03/12/2020 | Cerro Soto | Jaén | Alytes dickhilleni | 0.9 |
| TP2 | 10/12/2020 | Cerro Soto | Jaén | Alytes dickhilleni | 9.3 |
| TP2 | 10/12/2020 | Cerro Soto | Jaén | Alytes dickhilleni | 1 |
| TP2 | 10/12/2020 | Cerro Soto | Jaén | Alytes dickhilleni | 1 |
| TP2 | 10/12/2020 | Cerro Soto | Jaén | Alytes dickhilleni | 1 |
| TP2 | 10/12/2020 | Cerro Soto | Jaén | Alytes dickhilleni | 3.3 |
| TP2 | 10/12/2020 | Cerro Soto | Jaén | Alytes dickhilleni | 1.4 |
| TP2 | 10/12/2020 | Cerro Soto | Jaén | Alytes dickhilleni | 12.4 |
| TP2 | 10/12/2020 | Cerro Soto | Jaén | Alytes dickhilleni | 4.2 |
| TP3 | 17/12/2020 | Cerro Soto | Jaén | Alytes dickhilleni | 0 |
| TP3 | 17/12/2020 | Cerro Soto | Jaén | Alytes dickhilleni | 0 |
| TP3 | 17/12/2020 | Cerro Soto | Jaén | Alytes dickhilleni | 0 |
| TP3 | 17/12/2020 | Cerro Soto | Jaén | Alytes dickhilleni | 0 |
| TP3 | 17/12/2020 | Cerro Soto | Jaén | Alytes dickhilleni | 0 |
| TP3 | 17/12/2020 | Cerro Soto | Jaén | Alytes dickhilleni | 0 |
| TP3 | 17/12/2020 | Cerro Soto | Jaén | Alytes dickhilleni | 0.8 |
| TP3 | 17/12/2020 | Cerro Soto | Jaén | Alytes dickhilleni | 2.9 |
| TP3 | 17/12/2020 | Cerro Soto | Jaén | Alytes dickhilleni | 0.6 |
| TP3 | 17/12/2020 | Cerro Soto | Jaén | Alytes dickhilleni | 0 |
| TP3 | 17/12/2020 | Cerro Soto | Jaén | Alytes dickhilleni | 0.6 |
| TP3 | 17/12/2020 | Cerro Soto | Jaén | Alytes dickhilleni | 0.2 |
| TP4 | 04/07/2022 | Cerro Soto | Jaén | Alytes dickhilleni | 0.4 |
| TP4 | 04/07/2022 | Cerro Soto | Jaén | Alytes dickhilleni | 0.5 |
| TP4 | 04/07/2022 | Cerro Soto | Jaén | Alytes dickhilleni | 6.3 |
| TP4 | 04/07/2022 | Cerro Soto | Jaén | Alytes dickhilleni | 0.6 |
| TP4 | 04/07/2022 | Cerro Soto | Jaén | Alytes dickhilleni | 0.6 |
| TP4 | 04/07/2022 | Cerro Soto | Jaén | Alytes dickhilleni | 4.6 |
| TP4 | 04/07/2022 | Cerro Soto | Jaén | Alytes dickhilleni | 1.4 |
| TP4 | 04/07/2022 | Cerro Soto | Jaén | Alytes dickhilleni | 19.2 |
| TP1 | 30/06/2020 | Cortijo Coyote | Granada | Alytes dickhilleni | 0 |
| TP1 | 30/06/2020 | Cortijo Coyote | Granada | Alytes dickhilleni | 0 |
| TP1 | 30/06/2020 | Cortijo Coyote | Granada | Alytes dickhilleni | 0.8 |
| TP2 | 13/07/2020 | Cortijo Coyote | Granada | Alytes dickhilleni | 0 |
| TP2 | 13/07/2020 | Cortijo Coyote | Granada | Alytes dickhilleni | 0 |
| TP2 | 13/07/2020 | Cortijo Coyote | Granada | Alytes dickhilleni | 0 |
| TP2 | 13/07/2020 | Cortijo Coyote | Granada | Alytes dickhilleni | 0 |
| TP3 | 21/07/2020 | Cortijo Coyote | Granada | Alytes dickhilleni | 0 |
| TP3 | 21/07/2020 | Cortijo Coyote | Granada | Alytes dickhilleni | 0 |
| TP3 | 21/07/2020 | Cortijo Coyote | Granada | Alytes dickhilleni | 0 |
| TP1 | 05/03/2020 | Cortijo Herreras | Granada | Alytes dickhilleni | 1.3 |
| TP1 | 05/03/2020 | Cortijo Herreras | Granada | Alytes dickhilleni | 0.8 |
| TP1 | 05/03/2020 | Cortijo Herreras | Granada | Alytes dickhilleni | 36.8 |
| TP1 | 05/03/2020 | Cortijo Herreras | Granada | Alytes dickhilleni | 4 |
| TP1 | 05/03/2020 | Cortijo Herreras | Granada | Alytes dickhilleni | 1.1 |
| TP1 | 05/03/2020 | Cortijo Herreras | Granada | Alytes dickhilleni | 1.7 |
| TP1 | 05/03/2020 | Cortijo Herreras | Granada | Alytes dickhilleni | 2.4 |
| TP1 | 05/03/2020 | Cortijo Herreras | Granada | Alytes dickhilleni | 7.8 |
| TP1 | 05/03/2020 | Cortijo Herreras | Granada | Alytes dickhilleni | 0 |
| TP1 | 05/03/2020 | Cortijo Herreras | Granada | Alytes dickhilleni | 0.7 |
| TP2 | 13/03/2020 | Cortijo Herreras | Granada | Alytes dickhilleni | 1.2 |
| TP2 | 13/03/2020 | Cortijo Herreras | Granada | Alytes dickhilleni | 0 |
| TP2 | 13/03/2020 | Cortijo Herreras | Granada | Alytes dickhilleni | 0 |
| TP2 | 13/03/2020 | Cortijo Herreras | Granada | Alytes dickhilleni | 0 |
| TP2 | 13/03/2020 | Cortijo Herreras | Granada | Alytes dickhilleni | 0.2 |
| TP2 | 13/03/2020 | Cortijo Herreras | Granada | Alytes dickhilleni | 0 |
| TP2 | 13/03/2020 | Cortijo Herreras | Granada | Alytes dickhilleni | 0 |
| TP2 | 13/03/2020 | Cortijo Herreras | Granada | Alytes dickhilleni | 0 |
| TP2 | 13/03/2020 | Cortijo Herreras | Granada | Alytes dickhilleni | 3 |
| TP2 | 13/03/2020 | Cortijo Herreras | Granada | Alytes dickhilleni | 0 |
| TP2 | 13/03/2020 | Cortijo Herreras | Granada | Alytes dickhilleni | 0 |
| TP2 | 13/03/2020 | Cortijo Herreras | Granada | Alytes dickhilleni | 0 |
| TP2 | 13/03/2020 | Cortijo Herreras | Granada | Alytes dickhilleni | 0 |
| TP2 | 13/03/2020 | Cortijo Herreras | Granada | Alytes dickhilleni | 0 |
| TP2 | 13/03/2020 | Cortijo Herreras | Granada | Alytes dickhilleni | 0 |
| TP3 | 29/04/2020 | Cortijo Herreras | Granada | Alytes dickhilleni | 0 |
| TP3 | 29/04/2020 | Cortijo Herreras | Granada | Alytes dickhilleni | 0 |
| TP3 | 29/04/2020 | Cortijo Herreras | Granada | Alytes dickhilleni | 0 |
| TP3 | 29/04/2020 | Cortijo Herreras | Granada | Alytes dickhilleni | 0 |
| TP3 | 29/04/2020 | Cortijo Herreras | Granada | Alytes dickhilleni | 0 |
| TP3 | 29/04/2020 | Cortijo Herreras | Granada | Alytes dickhilleni | 0 |
| TP3 | 29/04/2020 | Cortijo Herreras | Granada | Alytes dickhilleni | 0 |
| TP3 | 29/04/2020 | Cortijo Herreras | Granada | Alytes dickhilleni | 0 |
| TP3 | 29/04/2020 | Cortijo Herreras | Granada | Alytes dickhilleni | 0 |
| TP3 | 29/04/2020 | Cortijo Herreras | Granada | Alytes dickhilleni | 0 |
| TP3 | 29/04/2020 | Cortijo Herreras | Granada | Alytes dickhilleni | 0 |
| TP3 | 29/04/2020 | Cortijo Herreras | Granada | Alytes dickhilleni | 0 |
| TP3 | 29/04/2020 | Cortijo Herreras | Granada | Alytes dickhilleni | 0 |
| TP3 | 29/04/2020 | Cortijo Herreras | Granada | Alytes dickhilleni | 0 |
| TP3 | 29/04/2020 | Cortijo Herreras | Granada | Alytes dickhilleni | 0 |
| TP4 | 29/06/2022 | Cortijo Herreras | Granada | Alytes dickhilleni | 1.7 |
| TP4 | 29/06/2022 | Cortijo Herreras | Granada | Alytes dickhilleni | 0 |
| TP4 | 29/06/2022 | Cortijo Herreras | Granada | Alytes dickhilleni | 0 |
| TP4 | 29/06/2022 | Cortijo Herreras | Granada | Alytes dickhilleni | 0 |
| TP4 | 29/06/2022 | Cortijo Herreras | Granada | Alytes dickhilleni | 0 |
| TP4 | 29/06/2022 | Cortijo Herreras | Granada | Alytes dickhilleni | 0 |
| TP4 | 29/06/2022 | Cortijo Herreras | Granada | Alytes dickhilleni | 0 |
| TP4 | 29/06/2022 | Cortijo Herreras | Granada | Alytes dickhilleni | 0 |
| TP4 | 29/06/2022 | Cortijo Herreras | Granada | Alytes dickhilleni | 7.8 |
| TP4 | 29/06/2022 | Cortijo Herreras | Granada | Alytes dickhilleni | 0 |
| TP1 | 23/01/2020 | Fuente Borriqueros | Málaga | Alytes dickhilleni | 4 |
| TP1 | 23/01/2020 | Fuente Borriqueros | Málaga | Alytes dickhilleni | 17.3 |
| TP1 | 23/01/2020 | Fuente Borriqueros | Málaga | Alytes dickhilleni | 59.6 |
| TP1 | 23/01/2020 | Fuente Borriqueros | Málaga | Alytes dickhilleni | 84.1 |
| TP1 | 23/01/2020 | Fuente Borriqueros | Málaga | Alytes dickhilleni | 120.8 |
| TP1 | 23/01/2020 | Fuente Borriqueros | Málaga | Alytes dickhilleni | 58.8 |
| TP1 | 23/01/2020 | Fuente Borriqueros | Málaga | Alytes dickhilleni | 208.7 |
| TP2 | 30/01/2020 | Fuente Borriqueros | Málaga | Alytes dickhilleni | 0 |
| TP2 | 30/01/2020 | Fuente Borriqueros | Málaga | Alytes dickhilleni | 0 |
| TP2 | 30/01/2020 | Fuente Borriqueros | Málaga | Alytes dickhilleni | 0 |
| TP2 | 30/01/2020 | Fuente Borriqueros | Málaga | Alytes dickhilleni | 0 |
| TP2 | 30/01/2020 | Fuente Borriqueros | Málaga | Alytes dickhilleni | 0 |
| TP2 | 30/01/2020 | Fuente Borriqueros | Málaga | Alytes dickhilleni | 0 |
| TP2 | 30/01/2020 | Fuente Borriqueros | Málaga | Alytes dickhilleni | 0 |
| TP2 | 30/01/2020 | Fuente Borriqueros | Málaga | Alytes dickhilleni | 0 |
| TP2 | 30/01/2020 | Fuente Borriqueros | Málaga | Alytes dickhilleni | 0 |
| TP2 | 30/01/2020 | Fuente Borriqueros | Málaga | Alytes dickhilleni | 0 |
| TP3 | 07/02/2020 | Fuente Borriqueros | Málaga | Alytes dickhilleni | 0 |
| TP3 | 07/02/2020 | Fuente Borriqueros | Málaga | Alytes dickhilleni | 0 |
| TP3 | 07/02/2020 | Fuente Borriqueros | Málaga | Alytes dickhilleni | 0 |
| TP3 | 07/02/2020 | Fuente Borriqueros | Málaga | Alytes dickhilleni | 0 |
| TP3 | 07/02/2020 | Fuente Borriqueros | Málaga | Alytes dickhilleni | 0 |
| TP3 | 07/02/2020 | Fuente Borriqueros | Málaga | Alytes dickhilleni | 0 |
| TP3 | 07/02/2020 | Fuente Borriqueros | Málaga | Alytes dickhilleni | 0 |
| TP3 | 07/02/2020 | Fuente Borriqueros | Málaga | Alytes dickhilleni | 0 |
| TP3 | 07/02/2020 | Fuente Borriqueros | Málaga | Alytes dickhilleni | 0 |
| TP3 | 07/02/2020 | Fuente Borriqueros | Málaga | Alytes dickhilleni | 0 |
| TP4 | 10/11/2020 | Fuente Borriqueros | Málaga | Alytes dickhilleni | 0 |
| TP4 | 10/11/2020 | Fuente Borriqueros | Málaga | Alytes dickhilleni | 0 |
| TP4 | 10/11/2020 | Fuente Borriqueros | Málaga | Alytes dickhilleni | 0 |
| TP4 | 10/11/2020 | Fuente Borriqueros | Málaga | Alytes dickhilleni | 0 |
| TP4 | 10/11/2020 | Fuente Borriqueros | Málaga | Alytes dickhilleni | 0 |
| TP4 | 10/11/2020 | Fuente Borriqueros | Málaga | Alytes dickhilleni | 0 |
| TP4 | 10/11/2020 | Fuente Borriqueros | Málaga | Alytes dickhilleni | 0 |
| TP4 | 10/11/2020 | Fuente Borriqueros | Málaga | Alytes dickhilleni | 0 |
| TP4 | 10/11/2020 | Fuente Borriqueros | Málaga | Alytes dickhilleni | 0 |
| TP1 | 17/03/2021 | Hoya de la Viga | Jaén | Alytes dickhilleni | 0 |
| TP1 | 17/03/2021 | Hoya de la Viga | Jaén | Alytes dickhilleni | 248 |
| TP1 | 17/03/2021 | Hoya de la Viga | Jaén | Alytes dickhilleni | 57 |
| TP1 | 17/03/2021 | Hoya de la Viga | Jaén | Alytes dickhilleni | 70.2 |
| TP1 | 17/03/2021 | Hoya de la Viga | Jaén | Alytes dickhilleni | 450 |
| TP1 | 17/03/2021 | Hoya de la Viga | Jaén | Alytes dickhilleni | 0 |
| TP1 | 17/03/2021 | Hoya de la Viga | Jaén | Alytes dickhilleni | 51.3 |
| TP1 | 17/03/2021 | Hoya de la Viga | Jaén | Alytes dickhilleni | 744.5 |
| TP1 | 17/03/2021 | Hoya de la Viga | Jaén | Alytes dickhilleni | 198.5 |
| TP2 | 24/03/2021 | Hoya de la Viga | Jaén | Alytes dickhilleni | 3.3 |
| TP2 | 24/03/2021 | Hoya de la Viga | Jaén | Alytes dickhilleni | 0.8 |
| TP2 | 24/03/2021 | Hoya de la Viga | Jaén | Alytes dickhilleni | 0.8 |
| TP2 | 24/03/2021 | Hoya de la Viga | Jaén | Alytes dickhilleni | 0 |
| TP2 | 24/03/2021 | Hoya de la Viga | Jaén | Alytes dickhilleni | 0.5 |
| TP2 | 24/03/2021 | Hoya de la Viga | Jaén | Alytes dickhilleni | 0.3 |
| TP2 | 24/03/2021 | Hoya de la Viga | Jaén | Alytes dickhilleni | 11.4 |
| TP2 | 24/03/2021 | Hoya de la Viga | Jaén | Alytes dickhilleni | 6.4 |
| TP2 | 24/03/2021 | Hoya de la Viga | Jaén | Alytes dickhilleni | 1.3 |
| TP2 | 24/03/2021 | Hoya de la Viga | Jaén | Alytes dickhilleni | 8.3 |
| TP3 | 29/04/2021 | Hoya de la Viga | Jaén | Alytes dickhilleni | 0 |
| TP3 | 29/04/2021 | Hoya de la Viga | Jaén | Alytes dickhilleni | 0 |
| TP3 | 29/04/2021 | Hoya de la Viga | Jaén | Alytes dickhilleni | 0 |
| TP3 | 29/04/2021 | Hoya de la Viga | Jaén | Alytes dickhilleni | 0 |
| TP3 | 29/04/2021 | Hoya de la Viga | Jaén | Alytes dickhilleni | 0 |
| TP3 | 29/04/2021 | Hoya de la Viga | Jaén | Alytes dickhilleni | 0 |
| TP3 | 29/04/2021 | Hoya de la Viga | Jaén | Alytes dickhilleni | 0 |
| TP3 | 29/04/2021 | Hoya de la Viga | Jaén | Alytes dickhilleni | 0 |
| TP3 | 29/04/2021 | Hoya de la Viga | Jaén | Alytes dickhilleni | 0 |
| TP3 | 29/04/2021 | Hoya de la Viga | Jaén | Alytes dickhilleni | 0 |
| TP4 | 27/06/2022 | Hoya de la Viga | Jaén | Alytes dickhilleni | 0 |
| TP4 | 27/06/2022 | Hoya de la Viga | Jaén | Alytes dickhilleni | 0 |
| TP4 | 27/06/2022 | Hoya de la Viga | Jaén | Alytes dickhilleni | 0 |
| TP4 | 27/06/2022 | Hoya de la Viga | Jaén | Alytes dickhilleni | 0 |
| TP4 | 27/06/2022 | Hoya de la Viga | Jaén | Alytes dickhilleni | 0 |
| TP4 | 27/06/2022 | Hoya de la Viga | Jaén | Alytes dickhilleni | 0 |
| TP4 | 27/06/2022 | Hoya de la Viga | Jaén | Alytes dickhilleni | 0 |
| TP4 | 27/06/2022 | Hoya de la Viga | Jaén | Alytes dickhilleni | 0 |
| TP4 | 27/06/2022 | Hoya de la Viga | Jaén | Alytes dickhilleni | 0 |
| TP4 | 27/06/2022 | Hoya de la Viga | Jaén | Alytes dickhilleni | 0 |
| TP1 | 01/11/2019 | Lancas | Granada | Alytes dickhilleni | 0 |
| TP1 | 01/11/2019 | Lancas | Granada | Alytes dickhilleni | 0 |
| TP1 | 01/11/2019 | Lancas | Granada | Alytes dickhilleni | 0 |
| TP1 | 01/11/2019 | Lancas | Granada | Alytes dickhilleni | 0 |
| TP1 | 01/11/2019 | Lancas | Granada | Alytes dickhilleni | 0 |
| TP1 | 01/11/2019 | Lancas | Granada | Alytes dickhilleni | 0 |
| TP1 | 01/11/2019 | Lancas | Granada | Alytes dickhilleni | 0 |
| TP1 | 05/12/2019 | Lancas | Granada | Alytes dickhilleni | 856.9 |
| TP2 | 12/12/2019 | Lancas | Granada | Alytes dickhilleni | 0 |
| TP2 | 12/12/2019 | Lancas | Granada | Alytes dickhilleni | 0 |
| TP2 | 12/12/2019 | Lancas | Granada | Alytes dickhilleni | 0 |
| TP2 | 12/12/2019 | Lancas | Granada | Alytes dickhilleni | 0 |
| TP2 | 12/12/2019 | Lancas | Granada | Alytes dickhilleni | 0 |
| TP2 | 12/12/2019 | Lancas | Granada | Alytes dickhilleni | 0 |
| TP2 | 12/12/2019 | Lancas | Granada | Alytes dickhilleni | 0 |
| TP2 | 12/12/2019 | Lancas | Granada | Alytes dickhilleni | 0 |
| TP2 | 12/12/2019 | Lancas | Granada | Alytes dickhilleni | 0 |
| TP2 | 12/12/2019 | Lancas | Granada | Alytes dickhilleni | 0 |
| TP2 | 12/12/2019 | Lancas | Granada | Alytes dickhilleni | 0.2 |
| TP2 | 12/12/2019 | Lancas | Granada | Alytes dickhilleni | 0 |
| TP2 | 12/12/2019 | Lancas | Granada | Alytes dickhilleni | 4.6 |
| TP2 | 12/12/2019 | Lancas | Granada | Alytes dickhilleni | 0 |
| TP3 | 21/12/2019 | Lancas | Granada | Alytes dickhilleni | 0 |
| TP3 | 21/12/2019 | Lancas | Granada | Alytes dickhilleni | 0 |
| TP3 | 21/12/2019 | Lancas | Granada | Alytes dickhilleni | 0 |
| TP3 | 21/12/2019 | Lancas | Granada | Alytes dickhilleni | 0 |
| TP3 | 21/12/2019 | Lancas | Granada | Salamandra salamandra | 0 |
| TP3 | 21/12/2019 | Lancas | Granada | Salamandra salamandra | 0 |
| TP3 | 21/12/2019 | Lancas | Granada | Salamandra salamandra | 0 |
| TP4 | 17/12/2020 | Lancas | Granada | Salamandra salamandra | 0 |
| TP4 | 17/12/2020 | Lancas | Granada | Salamandra salamandra | 0 |
| TP4 | 29/06/2022 | Lancas | Granada | Alytes dickhilleni | 0 |
| TP4 | 29/06/2022 | Lancas | Granada | Alytes dickhilleni | 0 |
| TP4 | 29/06/2022 | Lancas | Granada | Alytes dickhilleni | 0 |
| TP4 | 29/06/2022 | Lancas | Granada | Alytes dickhilleni | 0 |
| TP4 | 29/06/2022 | Lancas | Granada | Alytes dickhilleni | 0 |
| TP4 | 29/06/2022 | Lancas | Granada | Alytes dickhilleni | 0 |
| TP4 | 29/06/2022 | Lancas | Granada | Alytes dickhilleni | 0 |
| TP4 | 29/06/2022 | Lancas | Granada | Alytes dickhilleni | 0 |
| TP4 | 29/06/2022 | Lancas | Granada | Alytes dickhilleni | 0 |
| TP1 | 10/07/2020 | Loma de la Matanza | Granada | Alytes dickhilleni | 175.7 |
| TP1 | 10/07/2020 | Loma de la Matanza | Granada | Alytes dickhilleni | 277.9 |
| TP1 | 10/07/2020 | Loma de la Matanza | Granada | Alytes dickhilleni | 0.2 |
| TP1 | 10/07/2020 | Loma de la Matanza | Granada | Alytes dickhilleni | 390.4 |
| TP1 | 10/07/2020 | Loma de la Matanza | Granada | Alytes dickhilleni | 60.3 |
| TP1 | 10/07/2020 | Loma de la Matanza | Granada | Alytes dickhilleni | 164.1 |
| TP1 | 10/07/2020 | Loma de la Matanza | Granada | Alytes dickhilleni | 173.7 |
| TP1 | 10/07/2020 | Loma de la Matanza | Granada | Alytes dickhilleni | 276.1 |
| TP1 | 10/07/2020 | Loma de la Matanza | Granada | Alytes dickhilleni | 158.2 |
| TP1 | 10/07/2020 | Loma de la Matanza | Granada | Alytes dickhilleni | 190.7 |
| TP2 | 16/07/2020 | Loma de la Matanza | Granada | Alytes dickhilleni | 0 |
| TP2 | 16/07/2020 | Loma de la Matanza | Granada | Alytes dickhilleni | 0.3 |
| TP2 | 16/07/2020 | Loma de la Matanza | Granada | Alytes dickhilleni | 0 |
| TP2 | 16/07/2020 | Loma de la Matanza | Granada | Alytes dickhilleni | 0 |
| TP2 | 16/07/2020 | Loma de la Matanza | Granada | Alytes dickhilleni | 0.3 |
| TP2 | 16/07/2020 | Loma de la Matanza | Granada | Alytes dickhilleni | 0 |
| TP2 | 16/07/2020 | Loma de la Matanza | Granada | Alytes dickhilleni | 0 |
| TP2 | 16/07/2020 | Loma de la Matanza | Granada | Alytes dickhilleni | 0.6 |
| TP2 | 16/07/2020 | Loma de la Matanza | Granada | Alytes dickhilleni | 0 |
| TP2 | 16/07/2020 | Loma de la Matanza | Granada | Alytes dickhilleni | 0 |
| TP3 | 21/07/2020 | Loma de la Matanza | Granada | Alytes dickhilleni | 0 |
| TP3 | 21/07/2020 | Loma de la Matanza | Granada | Alytes dickhilleni | 0 |
| TP3 | 21/07/2020 | Loma de la Matanza | Granada | Alytes dickhilleni | 0 |
| TP3 | 21/07/2020 | Loma de la Matanza | Granada | Alytes dickhilleni | 0 |
| TP3 | 21/07/2020 | Loma de la Matanza | Granada | Alytes dickhilleni | 0 |
| TP3 | 21/07/2020 | Loma de la Matanza | Granada | Alytes dickhilleni | 0 |
| TP3 | 21/07/2020 | Loma de la Matanza | Granada | Alytes dickhilleni | 0 |
| TP3 | 21/07/2020 | Loma de la Matanza | Granada | Alytes dickhilleni | 0 |
| TP3 | 21/07/2020 | Loma de la Matanza | Granada | Alytes dickhilleni | 0 |
| TP3 | 21/07/2020 | Loma de la Matanza | Granada | Alytes dickhilleni | 0 |
| TP4 | 06/07/2022 | Loma de la Matanza | Granada | Alytes dickhilleni | 14.9 |
| TP4 | 06/07/2022 | Loma de la Matanza | Granada | Pelophylax perezi | 9 |
| TP4 | 06/07/2022 | Loma de la Matanza | Granada | Pelophylax perezi | 0.4 |
| TP4 | 06/07/2022 | Loma de la Matanza | Granada | Pelophylax perezi | 2.2 |
| TP1 | 13/11/2020 | Peñoncillos | Granada | Alytes dickhilleni | 14.4 |
| TP1 | 13/11/2020 | Peñoncillos | Granada | Alytes dickhilleni | 1.9 |
| TP1 | 13/11/2020 | Peñoncillos | Granada | Alytes dickhilleni | 16.9 |
| TP1 | 13/11/2020 | Peñoncillos | Granada | Alytes dickhilleni | 10.3 |
| TP1 | 13/11/2020 | Peñoncillos | Granada | Alytes dickhilleni | 3 |
| TP1 | 13/11/2020 | Peñoncillos | Granada | Alytes dickhilleni | 8.5 |
| TP1 | 13/11/2020 | Peñoncillos | Granada | Alytes dickhilleni | 9.3 |
| TP1 | 13/11/2020 | Peñoncillos | Granada | Alytes dickhilleni | 0.7 |
| TP1 | 13/11/2020 | Peñoncillos | Granada | Alytes dickhilleni | 30.5 |
| TP2 | 23/11/2020 | Peñoncillos | Granada | Alytes dickhilleni | 0 |
| TP2 | 23/11/2020 | Peñoncillos | Granada | Alytes dickhilleni | 0.2 |
| TP2 | 23/11/2020 | Peñoncillos | Granada | Alytes dickhilleni | 0.9 |
| TP2 | 23/11/2020 | Peñoncillos | Granada | Alytes dickhilleni | 0.3 |
| TP2 | 23/11/2020 | Peñoncillos | Granada | Alytes dickhilleni | 0.8 |
| TP2 | 23/11/2020 | Peñoncillos | Granada | Alytes dickhilleni | 0.8 |
| TP3 | 02/12/2020 | Peñoncillos | Granada | Alytes dickhilleni | 1.5 |
| TP3 | 02/12/2020 | Peñoncillos | Granada | Alytes dickhilleni | 0.6 |
| TP3 | 02/12/2020 | Peñoncillos | Granada | Alytes dickhilleni | 0 |
| TP3 | 02/12/2020 | Peñoncillos | Granada | Alytes dickhilleni | 0 |
| TP3 | 02/12/2020 | Peñoncillos | Granada | Alytes dickhilleni | 0 |
| TP3 | 02/12/2020 | Peñoncillos | Granada | Alytes dickhilleni | 0 |
| TP3 | 02/12/2020 | Peñoncillos | Granada | Alytes dickhilleni | 0.8 |
| TP3 | 02/12/2020 | Peñoncillos | Granada | Alytes dickhilleni | 0 |
| TP3 | 02/12/2020 | Peñoncillos | Granada | Alytes dickhilleni | 0 |
| TP3 | 02/12/2020 | Peñoncillos | Granada | Alytes dickhilleni | 0.3 |
| TP3 | 02/12/2020 | Peñoncillos | Granada | Alytes dickhilleni | 0 |
| TP4 | 27/07/2022 | Peñoncillos | Granada | Alytes dickhilleni | 0 |
| TP4 | 27/07/2022 | Peñoncillos | Granada | Alytes dickhilleni | 0 |
| TP4 | 27/07/2022 | Peñoncillos | Granada | Alytes dickhilleni | 0 |
| TP4 | 27/07/2022 | Peñoncillos | Granada | Alytes dickhilleni | 0 |
| TP4 | 27/07/2022 | Peñoncillos | Granada | Alytes dickhilleni | 0 |
| TP4 | 27/07/2022 | Peñoncillos | Granada | Alytes dickhilleni | 0 |
| TP4 | 27/07/2022 | Peñoncillos | Granada | Alytes dickhilleni | 0 |
| TP4 | 27/07/2022 | Peñoncillos | Granada | Alytes dickhilleni | 0 |
| TP4 | 27/07/2022 | Peñoncillos | Granada | Alytes dickhilleni | 0 |
| TP4 | 27/07/2022 | Peñoncillos | Granada | Alytes dickhilleni | 0 |
| TP1 | 17/03/2021 | Roblehondo | Jaén | Alytes dickhilleni | 14.6 |
| TP1 | 17/03/2021 | Roblehondo | Jaén | Alytes dickhilleni | 43.9 |
| TP1 | 17/03/2021 | Roblehondo | Jaén | Alytes dickhilleni | 32.1 |
| TP1 | 17/03/2021 | Roblehondo | Jaén | Alytes dickhilleni | 23.9 |
| TP1 | 17/03/2021 | Roblehondo | Jaén | Alytes dickhilleni | 42.5 |
| TP1 | 17/03/2021 | Roblehondo | Jaén | Alytes dickhilleni | 1143.4 |
| TP1 | 17/03/2021 | Roblehondo | Jaén | Alytes dickhilleni | 0 |
| TP1 | 17/03/2021 | Roblehondo | Jaén | Alytes dickhilleni | 311.1 |
| TP1 | 17/03/2021 | Roblehondo | Jaén | Alytes dickhilleni | 24 |
| TP1 | 17/03/2021 | Roblehondo | Jaén | Alytes dickhilleni | 400.3 |
| TP1 | 17/03/2021 | Roblehondo | Jaén | Alytes dickhilleni | 6.8 |
| TP2 | 24/03/2021 | Roblehondo | Jaén | Alytes dickhilleni | 16.2 |
| TP2 | 24/03/2021 | Roblehondo | Jaén | Alytes dickhilleni | 0 |
| TP2 | 24/03/2021 | Roblehondo | Jaén | Alytes dickhilleni | 0.5 |
| TP2 | 24/03/2021 | Roblehondo | Jaén | Alytes dickhilleni | 19.5 |
| TP2 | 24/03/2021 | Roblehondo | Jaén | Alytes dickhilleni | 43.3 |
| TP2 | 24/03/2021 | Roblehondo | Jaén | Alytes dickhilleni | 2 |
| TP2 | 24/03/2021 | Roblehondo | Jaén | Alytes dickhilleni | 66.3 |
| TP2 | 24/03/2021 | Roblehondo | Jaén | Alytes dickhilleni | 0 |
| TP2 | 24/03/2021 | Roblehondo | Jaén | Alytes dickhilleni | 20.7 |
| TP2 | 24/03/2021 | Roblehondo | Jaén | Alytes dickhilleni | 0.9 |
| TP2 | 24/03/2021 | Roblehondo | Jaén | Alytes dickhilleni | 1.5 |
| TP3 | 29/04/2021 | Roblehondo | Jaén | Alytes dickhilleni | 0 |
| TP3 | 29/04/2021 | Roblehondo | Jaén | Alytes dickhilleni | 0 |
| TP3 | 29/04/2021 | Roblehondo | Jaén | Alytes dickhilleni | 0 |
| TP3 | 29/04/2021 | Roblehondo | Jaén | Alytes dickhilleni | 0 |
| TP3 | 29/04/2021 | Roblehondo | Jaén | Alytes dickhilleni | 0 |
| TP3 | 29/04/2021 | Roblehondo | Jaén | Alytes dickhilleni | 0 |
| TP3 | 29/04/2021 | Roblehondo | Jaén | Alytes dickhilleni | 0 |
| TP3 | 29/04/2021 | Roblehondo | Jaén | Alytes dickhilleni | 0 |
| TP3 | 29/04/2021 | Roblehondo | Jaén | Alytes dickhilleni | 0 |
| TP3 | 29/04/2021 | Roblehondo | Jaén | Alytes dickhilleni | 0 |
| TP4 | 27/06/2022 | Roblehondo | Jaén | Alytes dickhilleni | 0 |
| TP4 | 27/06/2022 | Roblehondo | Jaén | Alytes dickhilleni | 0 |
| TP4 | 27/06/2022 | Roblehondo | Jaén | Alytes dickhilleni | 0 |
| TP4 | 27/06/2022 | Roblehondo | Jaén | Alytes dickhilleni | 0 |
| TP4 | 27/06/2022 | Roblehondo | Jaén | Alytes dickhilleni | 0 |
| TP4 | 27/06/2022 | Roblehondo | Jaén | Alytes dickhilleni | 0 |
| TP4 | 27/06/2022 | Roblehondo | Jaén | Alytes dickhilleni | 0 |
| TP4 | 27/06/2022 | Roblehondo | Jaén | Alytes dickhilleni | 0 |
| TP4 | 27/06/2022 | Roblehondo | Jaén | Alytes dickhilleni | 0 |
| TP4 | 27/06/2022 | Roblehondo | Jaén | Alytes dickhilleni | 0 |
| TP4 | 27/06/2022 | Roblehondo | Jaén | Alytes dickhilleni | 0 |
